# Characteristic MRI Findings of a Scrotal Schwannoma: A Case Report

**DOI:** 10.7759/cureus.98754

**Published:** 2025-12-08

**Authors:** Lingbo Deng, Licheng Qiu, Xiang Liu

**Affiliations:** 1 Department of Medical Imaging, Peking University Shenzhen Hospital, Shenzhen, CHN; 2 Department of Radiology, Sun Yat-Sen Memorial Hospital, Sun Yat-Sen University, Guangzhou, CHN

**Keywords:** case report, diagnostic radiology, magnetic resonance imaging, nerve sheath tumor, paratesticular mass, scrotal schwannoma

## Abstract

Scrotal schwannomas are rare benign tumors that are frequently misdiagnosed preoperatively as more common paratesticular lesions. MRI offers superior soft-tissue characterization, which can provide key diagnostic clues. We present a case of a 25-year-old male patient with a 12-year history of a scrotal mass that showed significant enlargement over the past two years. Initial scrotal ultrasound identified a solid paratesticular mass, prompting multi‑parametric MRI for detailed tissue characterization given the indeterminate nature of the lesion. Marked hyperintensity on T2-weighted imaging with a central hypointense area (the "target sign"), iso-intensity on T1-weighted imaging, and progressive heterogeneous enhancement on dynamic post-contrast sequences. These findings were highly suggestive of a benign nerve sheath tumor. The lesion was surgically excised, and histopathological examination confirmed the diagnosis of a classic schwannoma. This case underscores the pivotal role of MRI in the diagnostic workup of unusual paratesticular masses. Recognition of characteristic features, particularly the T2 "target sign" in conjunction with the specific enhancement pattern, can allow radiologists to confidently suggest the diagnosis of scrotal schwannoma preoperatively, thereby facilitating optimal surgical planning and patient counseling.

## Introduction

The evaluation of scrotal masses primarily relies on clinical examination and high-frequency ultrasonography. While most cases are diagnosed as common conditions such as epididymal cysts or testicular tumors, rare pathologies, including paratesticular schwannomas, present considerable diagnostic challenges [1]. Schwannomas are benign tumors derived from Schwann cells, and their occurrence within the scrotum is extremely rare, with only a limited number of cases reported in the literature [2].

Although ultrasonography is the primary imaging modality, its ability to differentiate schwannomas from other solid paratesticular tumors can be limited due to overlapping sonographic features. This often leads to diagnostic uncertainty. In this context, multi-parametric MRI plays a crucial role by providing superior soft-tissue contrast and detailed morphological information. Specific MRI features, such as a well-circumscribed margin, characteristically heterogeneous high T2 signal intensity (sometimes exhibiting a central 'target sign'), and progressive heterogeneous enhancement, are highly valuable for suggesting a preoperative diagnosis of benign schwannoma and distinguishing it from other entities [3]. This case report aims to demonstrate these characteristic MRI findings in a histopathologically confirmed paratesticular schwannoma, illustrating how MRI resolves diagnostic uncertainty and can guide appropriate clinical management.

## Case presentation

**Figure 1:**
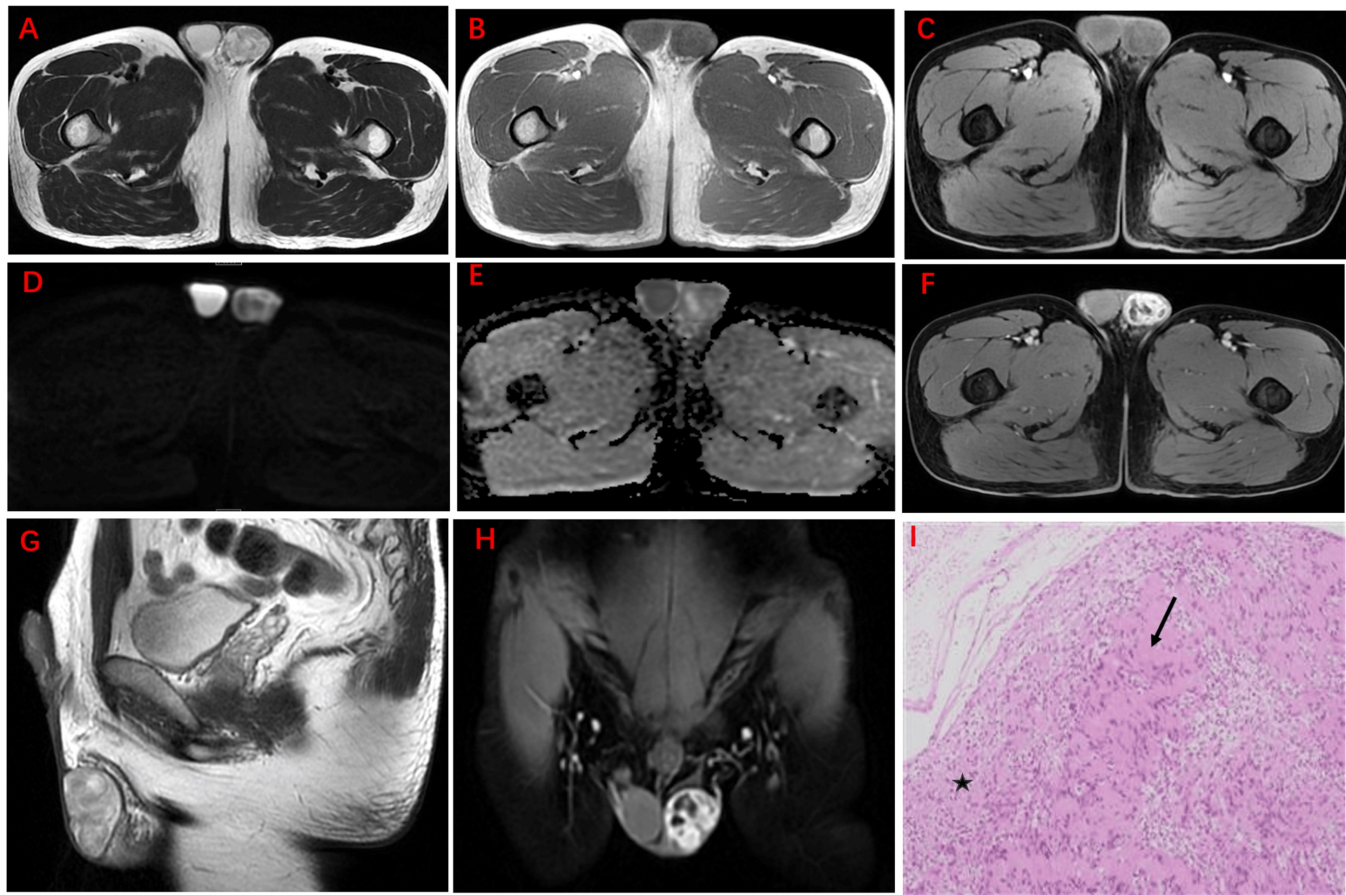
Paratesticular schwannoma in a 25-year-old male patient Figures 1A, 1G: T2-weighted imaging demonstrated a well-circumscribed, ovoid mass measuring 4 cm in maximum diameter. The lesion showed heterogeneous T2 signal intensity, containing scattered linear and patchy areas of low signal within a predominantly hyperintense background. Figures 1B, 1C: On T1-weighted imaging, the mass appeared homogeneously isointense to skeletal muscle. Figures 1D, 1E: Diffusion-weighted imaging revealed high signal intensity on high b-value images with corresponding moderately low signal on the apparent diffusion coefficient map, indicating restricted diffusion. Figures 1F, 1H: Dynamic contrast-enhanced MRI was done; following gadolinium administration, the lesion showed marked heterogeneous enhancement. The enhancement pattern correlated with T2 signal characteristics, demonstrating intense enhancement in T2-hypointense regions and only mild or no enhancement in T2-hyperintense areas. Figure 1I: Histopathological examination of the resected specimen confirmed the diagnosis of a benign schwannoma, characterized by classic alternating Antoni A and Antoni B areas.

A 25-year-old married male presented with a scrotal mass that had been present for 12 years. The mass demonstrated slow growth historically, with notable progression over the preceding two years. The patient, who had no children, reported no associated symptoms. An initial scrotal ultrasound performed at an outside facility identified a solid intrascrotal lesion. Physical examination revealed a firm, mobile nodule superior to the left testis, with no overlying skin erythema, warmth, or tenderness. Serum tumor markers (alpha-fetoprotein, beta human chorionic gonadotropin, and lactate dehydrogenase) were within normal limits. Multi-parametric MRI was subsequently employed for further characterization of the mass. Multi-parametric MRI findings are presented in Figures 1A-1H.

This constellation of imaging findings was most consistent with a benign nerve sheath tumor, particularly given the smooth contour, lack of necrosis, and predictable T2 and enhancement characteristics that distinguish schwannoma from sarcomas. The patient subsequently underwent elective surgical excision of the left scrotal mass under general anesthesia at our institution. Intraoperatively, a well-circumscribed, round, nodular mass with a complete capsule was identified in the left scrotal region and was completely resected. Histopathological examination of the resected specimen confirmed the diagnosis of a benign schwannoma, characterized by classic alternating Antoni A and Antoni B areas (Figure 1I). Immunohistochemical staining demonstrated robust diffuse positivity for S-100 protein, providing definitive confirmation. The patient's postoperative course was uncomplicated, and follow-up to date has shown no evidence of tumor recurrence.

## Discussion

Scrotal tumors are relatively uncommon in clinical practice and can be classified based on their tissue origin and biological behavior. Benign tumors of cutaneous origin primarily include sebaceous cysts and dermoid cysts, while malignant variants comprise scrotal squamous cell carcinoma, Paget's disease, melanoma, and dermatofibrosarcoma protuberans. Mesenchymal-derived benign tumors encompass entities such as epididymal adenoma, spermatic cord leiomyoma, and lipoma, among others, with corresponding malignancies including sarcomas of the tunica vaginalis, leiomyosarcoma, and angiosarcoma. Additionally, primary testicular tumors consist of seminomas, Leydig cell tumors, and Sertoli cell tumors.

MRI offers unique advantages in the evaluation of scrotal tumors due to its superior soft tissue resolution. It not only clearly delineates the anatomical origin, morphological characteristics, and relationship with surrounding structures but also reveals the internal tissue composition through multi-parametric sequences. The present case of a scrotal schwannoma serves as an illustrative example of the MRI manifestations of this rare entity in an uncommon location. Regarding MRI characteristics, the heterogeneous signal intensity on T2-weighted imaging, combined with the heterogeneous enhancement pattern on post-contrast sequences, forms a highly valuable diagnostic clue. Pathologically, the T2 hypointense areas with prominent enhancement correspond to Antoni A areas (composed of dense fibrous tissue and fascicular structures), whereas the T2 hyperintense areas with mild or no enhancement correlate with Antoni B areas (loose myxoid tissue) [4,5]. The presence of these features in a well-circumscribed paratesticular mass strongly supports the diagnosis of a benign schwannoma. Further analysis of the multi-parametric MRI features reveals complementary evidence: T2 hyperintensity reflects the high water content of the myxoid components; restricted diffusion on diffusion-weighted imaging is often associated with the high cellular density of Antoni A areas; and the progressive heterogeneous enhancement pattern is characteristic of schwannomas, related to the differential distribution of contrast agent within the various tumor components [6]. These multi-sequence findings are mutually reinforcing, providing comprehensive imaging support for an accurate diagnosis.

The clinical significance of recognizing these features lies primarily in differential diagnosis. Common paratesticular tumors such as leiomyoma [7] typically do not exhibit marked T2 hyperintensity or a "target sign." In contrast, malignant tumors like sarcomas often demonstrate infiltrative margins, internal necrosis, and more rapid, heterogeneous enhancement [8]. The lesion’s well‑defined borders and characteristic T2 and enhancement heterogeneity support a benign diagnosis; distinguishing it from fibroma and leiomyoma relies on the presence of the T2 ‘target sign’ and progressive heterogeneous enhancement, which are not typical for those tumors.The characteristic findings on T2-weighted and contrast-enhanced sequences support the diagnosis of schwannoma.

It is worth noting that while restricted diffusion is often observed in the highly cellular Antoni A areas of schwannomas, this imaging finding is not exclusive to benign lesions. Various malignant tumors (such as certain sarcomas or hypercellular neoplasms) may also demonstrate similar restricted diffusion patterns. Therefore, in clinical diagnosis, a comprehensive evaluation of other imaging features is necessary, for instance, the well-defined borders, markedly high T2 signal with a "target sign," and progressive heterogeneous enhancement observed in this case, which are more indicative of benignity, to more accurately differentiate between benign and malignant pathologies.

In conclusion, a confident preoperative MRI diagnosis of schwannoma is clinically valuable because it supports nerve‑sparing surgery and prevents overly aggressive interventions such as orchiectomy. It guides urologists in selecting appropriate surgical strategies, enabling nerve-sparing techniques when feasible, and provides accurate prognostic information, particularly given that schwannomas in this location are almost invariably benign. Such precise preoperative assessment is crucial for optimizing patient outcomes and improving quality of life.

## Conclusions

MRI is indispensable for the characterization of indeterminate paratesticular masses. In cases of scrotal schwannoma, specific MRI findings, particularly the T2 "target sign" in conjunction with progressive contrast enhancement, can facilitate a confident preoperative diagnosis. Radiologist recognition of this distinct imaging signature is crucial for differentiating this rare entity from more common pathologies and for guiding appropriate clinical management.
