# Correction: Characteristic MRI Findings of a Scrotal Schwannoma: A Case Report

**DOI:** 10.7759/cureus.c387

**Published:** 2025-12-22

**Authors:** Lingbo Deng, Licheng Qiu, Xiang Liu

**Affiliations:** 1 Department of Medical Imaging, Peking University Shenzhen Hospital, Shenzhen, CHN; 2 Department of Radiology, Sun Yat-Sen Memorial Hospital, Sun Yat-Sen University, Guangzhou, CHN

The ethics statement of this article has been corrected to include the following: "This work was supported by the Shenzhen High-level Hospital Construction Fund and the Peking University Shenzhen Hospital Scientific Research Fund. The funders had no role in study design, data collection and analysis, decision to publish, or preparation of the manuscript."

